# A ultrasound-based radiomic approach to predict the nodal status in clinically negative breast cancer patients

**DOI:** 10.1038/s41598-022-11876-4

**Published:** 2022-05-12

**Authors:** Samantha Bove, Maria Colomba Comes, Vito Lorusso, Cristian Cristofaro, Vittorio Didonna, Gianluca Gatta, Francesco Giotta, Daniele La Forgia, Agnese Latorre, Maria Irene Pastena, Nicole Petruzzellis, Domenico Pomarico, Lucia Rinaldi, Pasquale Tamborra, Alfredo Zito, Annarita Fanizzi, Raffaella Massafra

**Affiliations:** 1 Struttura Semplice Dipartimentale Di Fisica Sanitaria, I.R.C.C.S. Istituto Tumori “Giovanni Paolo II”, Viale Orazio Flacco 65, 70124 Bari, Italy; 2 Unità Operativa Complessa Di Oncologia Medica, I.R.C.C.S. Istituto Tumori “Giovanni Paolo II”, Viale Orazio Flacco 65, 70124 Bari, Italy; 3 Dipartimento Di Medicina Di Precisione, Università Della Campania “Luigi Vanvitelli”, 80131 Napoli, Italy; 4 Struttura Semplice Dipartimentale Di Radiologia Senologica, I.R.C.C.S. Istituto Tumori “Giovanni Paolo II”, Viale Orazio Flacco 65, 70124 Bari, Italy; 5 Unità Operativa Complessa Di Anatomia Patologica, I.R.C.C.S. Istituto Tumori “Giovanni Paolo II”, Viale Orazio Flacco 65, 70124 Bari, Italy; 6 Struttura Semplice Dipartimentale Di Oncologia Per La Presa in Carico Globale del Paziente, I.R.C.C.S. Istituto Tumori “Giovanni Paolo II”, Viale Orazio Flacco 65, 70124 Bari, Italy

**Keywords:** Breast cancer, Metastasis

## Abstract

In breast cancer patients, an accurate detection of the axillary lymph node metastasis status is essential for reducing distant metastasis occurrence probabilities. In case of patients resulted negative at both clinical and instrumental examination, the nodal status is commonly evaluated performing the sentinel lymph-node biopsy, that is a time-consuming and expensive intraoperative procedure for the sentinel lymph-node (SLN) status assessment. The aim of this study was to predict the nodal status of 142 clinically negative breast cancer patients by means of both clinical and radiomic features extracted from primary breast tumor ultrasound images acquired at diagnosis. First, different regions of interest (ROIs) were segmented and a radiomic analysis was performed on each ROI. Then, clinical and radiomic features were evaluated separately developing two different machine learning models based on an SVM classifier. Finally, their predictive power was estimated jointly implementing a soft voting technique. The experimental results showed that the model obtained by combining clinical and radiomic features provided the best performances, achieving an AUC value of 88.6%, an accuracy of 82.1%, a sensitivity of 100% and a specificity of 78.2%. The proposed model represents a promising non-invasive procedure for the SLN status prediction in clinically negative patients.

## Introduction

Nowadays, about 98.6% of breast cancer female patients survive within 5 years after diagnosis. Nevertheless, this rate decreases to 84.4% in case of axillary lymph nodes (ALN) metastasis^1,2^. For this reason, a timely and careful diagnosis of the nodal status is extremely important, especially in patients resulted negative at both clinical and instrumental examination. In clinically negative patients, the first step in the ALN metastatic status prediction is the sentinel lymph node (SLN) evaluation by means of the sentinel lymph-node biopsy (SLNB). Subsequently, in case of SLN positive status, the guidelines provide the axillary lymph node dissection (ALND). With the aim of reducing the number of surgical procedures and the hospitalization, the ALND and the primary breast tumor surgery can be performed at the same time, thus, an earlier knowledge of the SLN status is necessary. One of the most effective techniques for the intraoperative detection of metastasis in SLN is the one-step nucleic acid amplification (OSNA). The OSNA is an high-performance procedure with an accuracy greater than 96%, a sensitivity of 87.5–100% and a specificity equals to 90.5–100%, nonetheless it is a time-consuming and expensive examination which could result in different side effects, such as allergic reactions, wound infection, seroma, paresthesia, lymphedema, and hematoma^3–6^. Furthermore, a specialist in nuclear medicine afferent to a nuclear medicine department, within the hospital or connected to it, is needed.

Therefore, the development of a non-invasive preoperative assessment of the SLN status is attracting growing attention^7^. So far, in the state-of-the-art, several studies have proposed reliable alternative to the SLNB for the nodal status prediction in breast cancer patients. Particularly, more recent works involved not only clinical factors, but also radiomic features extracted by primary breast tumor images acquired at diagnosis in different ways^8–10^. In the work of Yang et al.^8^ the authors developed a nomogram which combined clinical information with textural and shape features extracted from ROIs identified on presurgical mammography images. The approach proposed in the work of Santucci et al.^9^ aimed to predict the nodal status by combining histological features with radiomic features computed on ROIs extracted from 3 Tesla post contrast—magnetic resonance images. In the work of Liu et al.^10^ the authors evaluated the predictive power of radiomic features extracted from ROIs identified on dynamic contrast-enhanced magnetic resonance images.

Other research works performed a radiomic analysis on ROIs extracted from primary breast tumor ultrasound images acquired at diagnosis^11–14^. However, to the best of our knowledge, there are not studies which propose ultrasound radiomic-based model for the SLN status prediction in clinically negative breast cancer patients, that are patients characterized by an early stage breast cancer and whose nodal positivity is difficult to diagnose.

As a matter of fact, there are few models designed to clinically negative patients, and these are based on only clinical features^15–18^. In our previous work^17^, we used clinical and histopathological features to implement machine learning predictive models. Thus, to improve our previous results, in this study we developed a preoperative tool for the SLN metastatic status prediction in clinically negative patients, analyzing radiomic features extracted from primary tumor ultrasound images. Indeed, ultrasound represents the least expansive and invasive technique with respect to other diagnostic tools, such as mammography, magnetic resonance and contrast-enhanced devices. Particularly, after a radiomic analysis performed on different ROIs by means of four gray-level occurrence matrices, we compared different approaches which evaluated clinical and radiomic features, both individually and combined, to identify a proper model to replace the OSNA procedure without compromising the diagnosis accuracy.

## Results

### Data description and statistical analysis results

This retrospective study was approved by the Scientific Board of the Istituto Tumori “Giovanni Paolo II” of Bari, Italy, and only patients who gave consent to use the data were considered. Particularly, female patients with a first breast cancer diagnosis in the period 2017–2020 were recruited. The eligibility criteria were (a) patients resulted negative at both clinical and instrumental examination, (b) patients who underwent the one-step nucleic acid amplification (OSNA) procedure, (c) patients with known ALN metastatic status and (d) patients with primary tumor ultrasound images acquired at diagnosis. Finally, 142 patients, of which 115 with negative ALN metastatic status and 27 with positive ALN metastatic status, were included in this study. Notably, the dataset imbalance was due to the low incidence of axillary nodal metastasis in clinically negative patients.

For each patient, along with a primary breast tumor ultrasound image acquired at diagnosis, baseline clinical and histopathological data were collected, including age at diagnosis (abbr. age), tumor size (abbr. diameter, values: T1a, T1b, T1c, T2), histological grade (abbr. grading, values: 1, 2, 3), histological type (values: ductal, lobular, other types), estrogen receptor expression (abbr. ER, % value), progesterone receptor expression (abbr. PgR, % value), cellular marker for proliferation (abbr. ki67, % value), human epidermal growth factor receptor-2 score (abbr. HER2/neu, values: 0, 1, 2, 3), breast quadrant (abbr. quadrant, values: QSM, QSE, QEE, QIE, QIM, QII, QEI, QSI), tumor multifocality (abbr. multifocality, values: absent/present), angioinvasion (values: absent/present) and tumor invasiveness (abbr. invasiveness, values: infiltrating/in situ).

Thus, a set of 12 clinical features was obtained and the few missing data (*see* Table 1) were estimated by means of a proximity algorithm. For each patient with at least one missing clinical feature, the proximity technique allows to replace his missing data with data belonging to the patient without missing features and whose values had the minimum Euclidean distance from the patient under consideration^19^. Due to the exiguity of the sample and with the aim of improving the accuracy of the estimated data, the data imputation procedure was implemented on the whole dataset. Besides, potential missing features of newcome patients will be estimated comparing each incomplete observation to the 142 patients belonging to the dataset employed in this study.

**Table 1:** Clinical features distribution over the study population. The asterisk * highlights features with a *p*-value less than 0.05. The statistical analysis was performed by means of the Mann–Whitney test for variables measured on a continuous scale and the Chi-square test for variables measured on a nominal scale.

Feature	Distribution	Feature	Distribution
**Overall**	142; 100%	**Quadrant**	
Age *		QSM (abs.; %)	20; 14.1%
Median; [q_1_, q_3_]	60 [48, 69]	QSE (abs.; %)	46; 32.4%
NA (abs.; %)	13; 9.2%	QEE (abs.; %)	20; 14.1%
Diameter *		QIE (abs.; %)	15; 10.6%
T1a (abs.; %)	12; 8.5%	QIM (abs.; %)	1; 0.7%
T1b (abs.; %)	42; 29.6%	QII (abs.; %)	8; 5.6%
T1c (abs.; %)	57; 40.1%	QEI (abs.; %)	9; 6.3%
T2 (abs.; %)	30; 21.1%	QSI (abs.; %)	23; 16.2%
NA (abs.; %)	1; 0.7%	**PgR**	
**Grading**		Median; [q_1_, q_3_]	40 [5, 80]
G1 (abs.; %)	40; 28.2%	NA (abs.; %)	1; 0.7%
G2 (abs.; %)	62; 43.7%	**ki67**	
G3 (abs.; %)	38; 26.7%	Median; [q_1_, q_3_]	18 [12, 30]
NA (abs.; %)	2; 1.4%	NA (abs.; %)	2; 1.4%
**Histological type**		**Her2/neu**	
Ductal (abs.; %)	115; 80.9%	0 (abs.; %)	87; 61.3%
Lobular (abs.; %)	21; 14.9%	1 (abs.; %)	30; 21.1%
Others (abs.; %)	4; 2.8%	2 (abs.; %)	13; 9.2%
NA (abs.; %)	2; 1.4%	3 (abs.; %)	10; 7.0%
**ER**		NA (abs.; %)	2; 1.4%
Median; [q_1_, q_3_]	98 [95, 98]	Multifocality *	
NA (abs.; %)	1; 0.7%	Absent (abs.; %)	110; 77.5%
**Invasiveness**		Present (abs.; %)	32; 22.5%
Infiltrating (abs.; %)	129; 90.8%	Angioinvasion *	
In situ (abs.; %)	11; 7.8%	Absent (abs.; %)	121; 85.2%
NA (abs.; %)	2; 1.4%	Present (abs.; %)	21; 14.8%

An overview about the sample properties is provided by Table 1. Features resulted discriminant in our statistical analysis (with a *p*-value less than 0.05), namely age, diameter, multifocality and angioinvasion, are highlighted. All the other variables did not show a statistically significant association with the ALN metastatic status. Nevertheless, since in our preliminary analysis the only statistically significant features resulted not discriminant in the outcome prediction, all the clinical features were included in the classification model. Interim results were not reported in order to not burden the discussion.

### Classification performances

Besides the clinical set, four radiomic sets were defined for each patient. Specifically, the radiomic analysis was performed on four different ROIs, namely, original ROI, intra-tumoral ROI, peritumoral ROI and combined ROI, extracted from the primary breast tumor ultrasound image acquired at diagnosis (*see* “Method”). Thus, a total of eleven learning models was developed to evaluate the predictive power of clinical and radiomic features, first individually and then simultaneously.

For each developed classification model, the hold-out training set consisted of 80% of the input sample, that is 114 randomly selected patients out of which 92 with negative ALN metastatic status and 22 with positive ALN metastatic status. Consequently, the hold-out test set, made up of the remaining 20% of the sample, included 28 patients of which 23 with negative ALN metastatic status and 5 with positive ALN metastatic status. Thus, the same percentage of positive patients was selected for both sets by performing the splitting procedure in training and test set according to a random stratified sampling. To proof the robustness of the performed splitting, in a supplementary table (Table S1) we reported the distributions of both sub-sets with respect to all clinical features involved in this study. The statistical analysis returned a p-value greater than 0.05 for ten of the twelve features, confirming the homogeneity which occurs between the training and test sets.

Classification performances achieved by all models on the hold-out training set are summarized in a supplementary table (Table S2) to not burden the discussion.

Meanwhile, Table 2 summarizes the classification performances achieved by all models on the hold-out test set. For each metric we specified the 95% confidence interval computed with the R library *pROC*. Each radiomic-based model is denoted by the name of the ROI from which the features were extracted (e.g., *Radiomic original* refers to the model obtained considering radiomic features extracted from the original ROI). Moreover, while the *Radiomic comb* model was designed extracting features from the combined ROIs, the last reported radiomic-based model *Radiomic intra* + *peri* refers to the model developed by merging the intra-tumoral and peritumoral datasets.

**Table 2:** Classification performances of all models on the hold-out test set. For each radiomic feature set, the performances of both the related radiomic-based model and the soft voting-based model are reported. The best result is highlighted in bold. Statistical quantifications were demonstrated with 95% confidential interval (CI), when applicable. The asterisk * highlights models whose AUC resulted statistically significant with a p-value less than 0.05.

	AUC (%)	Accuracy (%)	Sensitivity (%)	Specificity (%)
Clinical	73.9 (49–98)	82.1	60 (40–100)	86.9 (55–100)
Radiomic original *	75.6 (53–99)	67.8	80 (60–100)	65.2 (46.9–85.6)
Clinical/Radiomic original (SV) *	**88.6 (77–100)**	**82.1**	**100 (80–100)**	**78.2 (65–96)**
Radiomic intra	66.3 (45–88)	75	60 (40–100)	78.2 (45–86)
Clinical/Radiomic intra (SV) *	65.4 (44–89)	64.2	60 (40–100)	65.2 (46–81)
Radiomic peri	67.8 (48–87)	75	60 (40–100)	78.2 (61–100)
Clinical/Radiomic peri (SV) *	72.1 (51–100)	67.8	60 (40–100)	69.5 (48–78)
Radiomic comb	69.5 (46–93)	67.8	60 (40–100)	69.5 (45–81)
Clinical/Radiomic comb (SV) *	69.5 (46–99)	64.2	80 (60–100)	60.8 (47–91)
Radiomic intra + peri	66.3 (47–92)	75	60 (40–100)	78.2 (59–100)
Clinical/Radiomic intra + peri (SV) *	78.1 (55–99)	78.5	60 (40–100)	82.6 (55–100)

**Figure 1:**
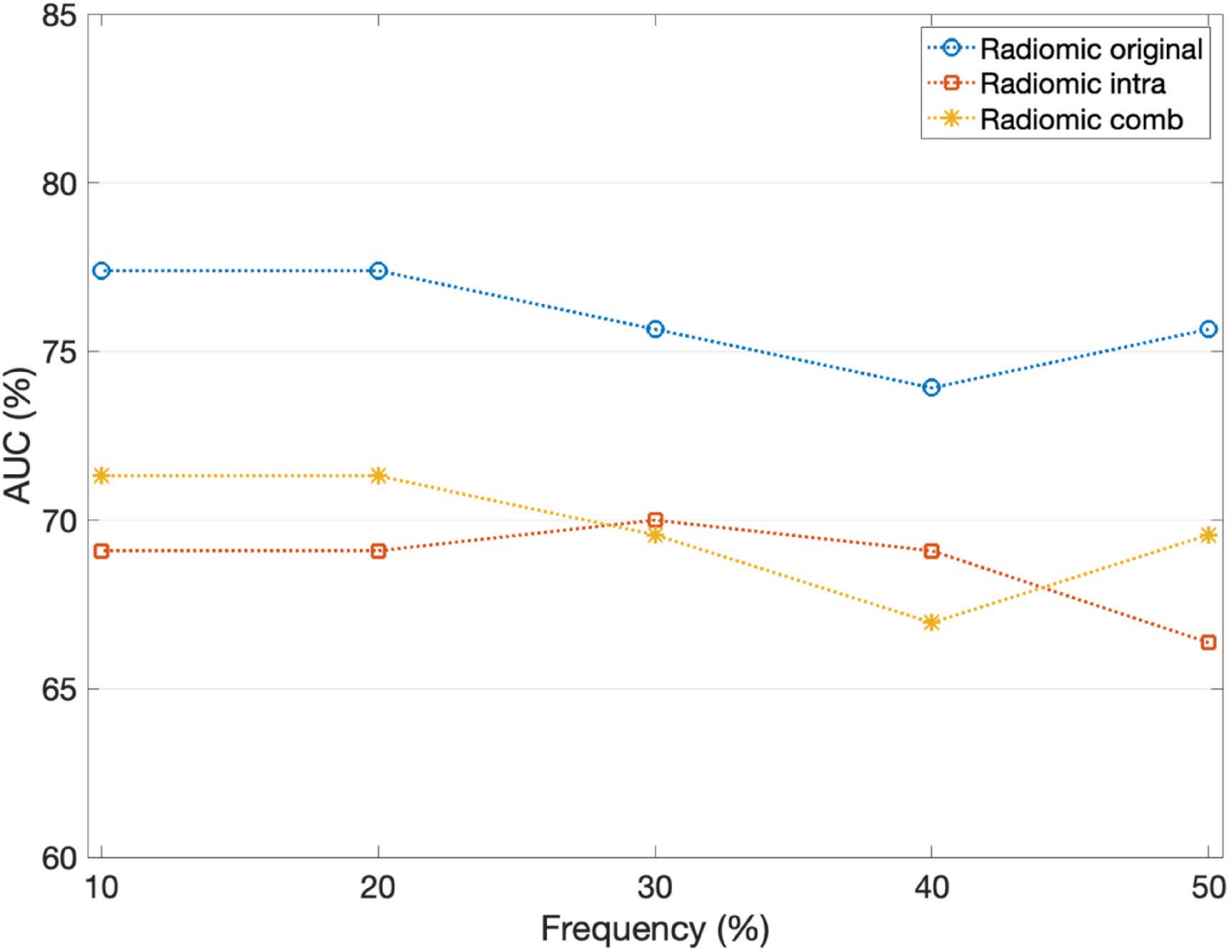
AUC distributions of some radiomic-based models on the hold-out test set by decreasing the number of features according to the feature selection frequency over the leave-one-out cross-validation rounds on the training set.

As regards to all radiomic-based models, the reported performances refer to the classification models trained on the sub-set of features obtained considering only the features selected at least 50% of the time over the leave-one-out cross-validation rounds on the training set. As a matter of fact, this represents the best trade-off between high performances and low-dimensional datasets: as some examples in Fig. 1 show, the AUC values do not undergo large variations as the dataset dimension decreases, that is, as the feature selection frequency increases. To be fair, since 50% was the greatest common frequency among all models, frequencies greater than 50% were not considered. The same criterion was applied for the soft voting-based models’ development.

The model developed by means of the only clinical features reached an AUC value of 73.9%, an accuracy of 82.1%, a sensitivity of 60% and a specificity of 86.9%. Regarding the radiomic-based models, rather, the best one in terms of AUC values resulted the *Radiomic original* one, which achieved an AUC value equals to 75.6%, an accuracy equals to 67.8%, a sensitivity and a specificity equal to 80% and 65.2%, respectively. Furthermore, radiomic features extracted from the original ROI resulted the most significant even when evaluated in association with the clinical features within the soft voting (SV) approach. Particularly, the *Clinical/Radiomic original (SV)* model reached an AUC value of 88.6%, an accuracy of 82.1%, a sensitivity of 100% and a specificity of 78.2%.

Further models were developed during the experimental phase of data analysis, for predicting the SLN status directly combining clinical and radiomic features. Nonetheless, since the performances achieved were not satisfying, we only reported in a supplementary table (Table S3) the results obtained by the model which directly combined radiomic features extracted from the original ROI and clinical features (Clinical + Radiomic original), and we compare these results with those achieved by the Clinical/Radiomic original (SV) model to demonstrate the greater effectiveness of the SV approach.

## Discussion

An early and accurate detection of the SLN metastatic status is essential not only to optimally treat breast cancer patients from a surgical point of view, but even to reduce the recurrence and/or distant metastasis occurrence probabilities. Nowadays, the guidelines provide for the one-step nucleic acid amplification (OSNA) as the intra-operative method for the SLN metastatic status detection in clinically negative breast cancer patients. The OSNA procedure achieves high performances, nevertheless it is a time-consuming and expansive examination which could lead to several side effects^3^. Furthermore, since the percentage of clinically negative patients which develop nodal metastasis is approximatively 15%, this could be an unnecessary invasive procedure.

Thus, the aim of this work was to devise a model able to predict the SLN metastatic status in clinically negative breast cancer patients, for proposing a non-invasive alternative to the OSNA procedure. Firstly, 142 clinically negative breast cancer patients which underwent the OSNA in our Institute were recruited. For each patient, a primary tumor ultrasound image acquired at diagnosis and several clinical features, such as age, diameter, grading, histological type, ER, PgR, ki67, HER2/neu, quadrant, multifocality, angioinvasion and invasiveness, were collected. Then, from each ultrasound image, four different frames, namely original ROI, intra-tumoral ROI, peri-tumoral ROI and combined ROI, were extracted and their texture was analyzed by means of radiomic features computed on four different gray-level occurrence matrices (*see* “Method”). Finally, eleven classification models based on an SVM classifier were developed, and their performances were evaluated on a hold-out test set in terms of AUC, accuracy, sensitivity, and specificity. All the radiomic-based models, additionally, were trained on the sub-set of features selected by a genetic algorithm within a leave-one-out cross-validation procedure on the training set (*see* “Method”).

The *Clinical* model reached an AUC, an accuracy, a sensitivity, and a specificity equal to73.9%, 82.1%, 60% and 86.9%, respectively. Among the radiomic-based models, the best performing in terms of AUC values was the model developed by means of the radiomic features extracted from the original ROI. Particularly, the *Radiomic original* model achieved an AUC of 75.6%, an accuracy of 67.8%, a sensitivity of 80% and a specificity of 65.2%. This model turned out to be the optimal choice also when evaluated in association with the clinical-based model within the soft voting approach. The *Clinical-Radiomic original (SV)* model, indeed, resulted the best one reaching an AUC of 88.6%, an accuracy of 82.1%, a sensitivity of 100%, and a specificity of 78.2%. Comparing these results with those obtained on the intra-tumoral ROI, it can be noticed that the influence of the peritumoral region in the original ROI provides discriminant information for the ALN metastatic status prediction. Furthermore, we can notice that the radiomic model on the original ROI outperforms the radiomic model intra + peri. These findings may be justified by the fact that the original ROI captures a large zone of peritumoral tissue, i.e., the tissue connecting between tumor and normal tissue, which has been demonstrated to be a site of tumor proliferation and angiogenesis^20^.Thus, the related extracted radiomic characteristics might higher correlated with the metastatic nodal status to be predicted. As a matter of fact, a statistical analysis proved the only statistically significant radiomic-based model resulted the one which exploited radiomic features extracted from the original ROI.These results are in agreement with research studies related to evaluation of tumor stiffness on US elastography with axillary nodal metastasis in early-stage breast cancer patients, where tumor stiffness was computed as the displacement of each pixel relative to the surroundings and converted into a colour display in real time^21^. Accordingly, in our future works we will investigate the nodal status prediction at varying the thickness of the peritumoral regions within the extracted ROIs.

The proposed model outperforms the ones developed in our previous works by means of the only clinical features. Indeed, in our first work Fanizzi et al.^16^ , an AUC value of 68%, an accuracy of 50.5%, a sensitivity of 69.8% and a specificity of 45.5% were achieved, on an independent test, considering the following features: diameter, age, histological type, grading, ER, PgR, ki67 and HER2. Comparable results were reached in our succeeding work Fanizzi et al.^17^, implementing a machine learning approach on the same clinical features, with the addition of two histological characteristics, such as multifocality and in-situ component. Our choice of reporting the performances of all investigated models had the purpose of highlighting the fundamental role of radiomic features for improving the performances of clinical-based predictive models. These results are consistent with the role of radiomics debated in the state-of-the-art^22^. Specifically, the ultrasound central role in the development of such a promising radiomic model for the nodal status prediction increases the importance of this non-invasive and inexpensive diagnostic procedure, especially as the dependence on the operator could be overcome through automatic acquisition system evaluations ^23^.

Our performances exceed also the results obtained by authors in the work of Dihge et al.^15^. They developed different artificial neural network-based models for the nodal status prediction in clinically negative patients by means of both clinical and radiological features, reaching a best AUC value of 74%.

An overwiew of the performances achieved by state-of-the-art models designed to clinically negative breast cancer patients is provived by Table 3.

**Table 3:** Nodal status prediction in clinically negative breast cancer patients: comparison among the state-of-the-art models performances.

	N. of patients	Model	Performances (%)
Fanizzi et al.^16^	993	Clinical-based	AUC 68.0
			Acc 50.5
			Sens 69.8
			Spe 45.5
Fanizzi et al.^17^	907	Clinical-based	Acc 62.1
			Sens 68.3
			Spe 59.7
Dihge et al.^15^	995	Artificial neural network-based	AUC 74
Best model proposed	142	Clinical/Radiomic-based	AUC 88.6
			Acc 82.1
			Sens 100
			Spe 78.2

While the above-mentioned approaches involved only clinical features, other approaches were developed considering also features extracted from ultrasound images by different techniques^11–14^.

In the work of Qiu et al.^11^ the authors extracted radiomic features from ultrasound images belonged to 196 breast cancer patients and developed a nomogram which achieved an AUC of 75.9% in the validation cohorts. Similarly, in the work of Zhou et al.^12^ the authors analyzed radiomic features extracted from ultrasound images of 192 breast cancer patients, reaching an AUC equals to 65% on the test set.

The approach proposed in the work of Sun et al.^13^ compares the performances achieved by radiomic-based and deep learning-based models implemented on features extracted from ultrasound images of 479 patients. Specifically, the images the authors used correspond to the intra-tumoral, peritumoral and combined images of our study. On the validation cohorts they obtained the best results analyzing the combined ROI, reaching an AUC equals to 83.3% with the radiomic-based model and an AUC value of 95% with the deep-learning one. As well as in our analysis, comparing the best results to the ones obtained by authors on the intra-tumoral ROI, the discriminant role of the peritumoral region in the ALN status prediction is confirmed.

A further deep learning-based model was developed in the work of Zheng et al.^14^. The authors achieved an AUC value of 90.2% combining clinical parameters and deep learning features extracted from ultrasound images belonged to 584 breast cancer patients.

Although our model is not yet suitable for clinical practice due to the low specificity and the exiguity of the sample, it represents an improvement of the state of art. Indeed, even if the last above-discussed models reached high performances, our approach is the only radiomic-based model designed for clinically negative breast cancer patients. Furthermore, our model is capable of correctly identifying all the metastatic sentinel lymph nodes, despite the greater difficulty in diagnosing the nodal positivity in clinically negative patients, that are patients characterized by an early stage breast cancer. Thus, it is a promising and inexpensive method which could replace the OSNA procedure without compromising the patient care.

Anyway, encouraged by the better results commonly achieved by deep learning-based models developed in the state-of-the-art within the ultrasound analysis framework^13,14^, in our future work we will devise a deep learning approach for predicting SLN metastatic status in clinically negative breast cancer patients, with the aim of improving the specificity starting from both the clinical features and the ultrasound images acquired at diagnosis.

## Method

### Ultrasound image acquisition and pre-processing

**Figure 2:**
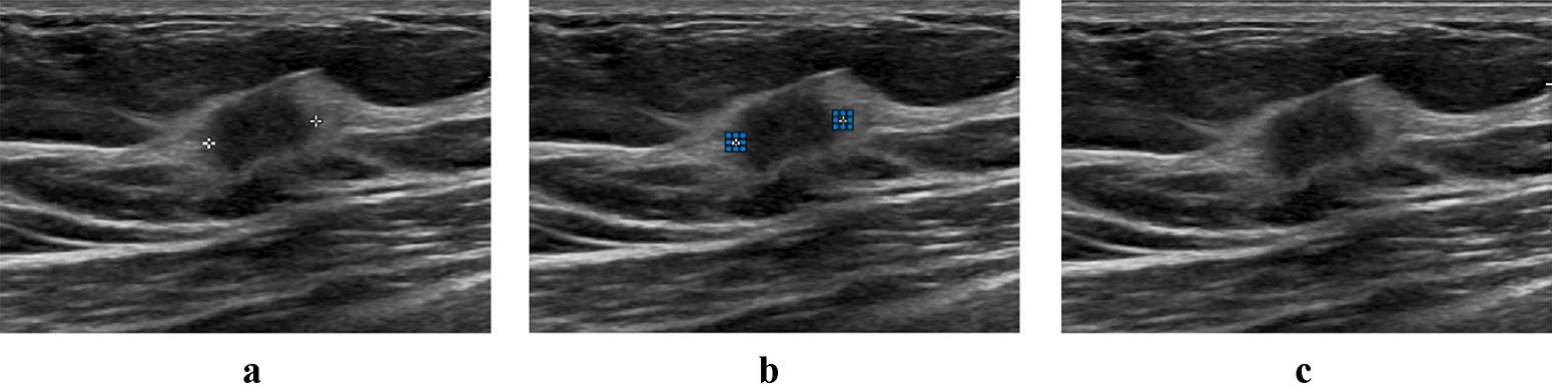
Ultrasound image inpainting process. Given the input image **(a)** and selected one or more target regions **(b)**, these are removed and replaced through a standard impainting technique **(c).**

Ultrasound scans were performed by means of a PHILIPS Affiniti 70 ultrasound system with L12-5 50 linear probe [5—12 MHz] 256 elements, 50 mm, fine pitch. Subsequently, ultrasound images enclosing the neoplastic lesion were retrospectively acquired by the Picture Archiving and Communication System (PACS), and for each patient an image was selected by a breast radiologist from our Institute with more than 20 years of experience. As depicted in Fig. 2, since in each image the region of interest was delimited by markers during the screening phase, an inpainting technique was implemented for these objects’ removal and replacement. In detail, given an input image and selected a target region, this technique allows to remove pixels in this region and replace them using an exemplar-based texture synthesis, namely, the process of generating a new texture image perceptively equals to the input sample^24^. In this study, an inpainting technique based on coherence transport was adopted. Defined the inpainting domain, the coherence transport permits to propagate image values according to a transport equation in a coherent direction, that is the direction along which a specified degree of coherence among gray-level values is maintained^25^.

**Figure 3:**
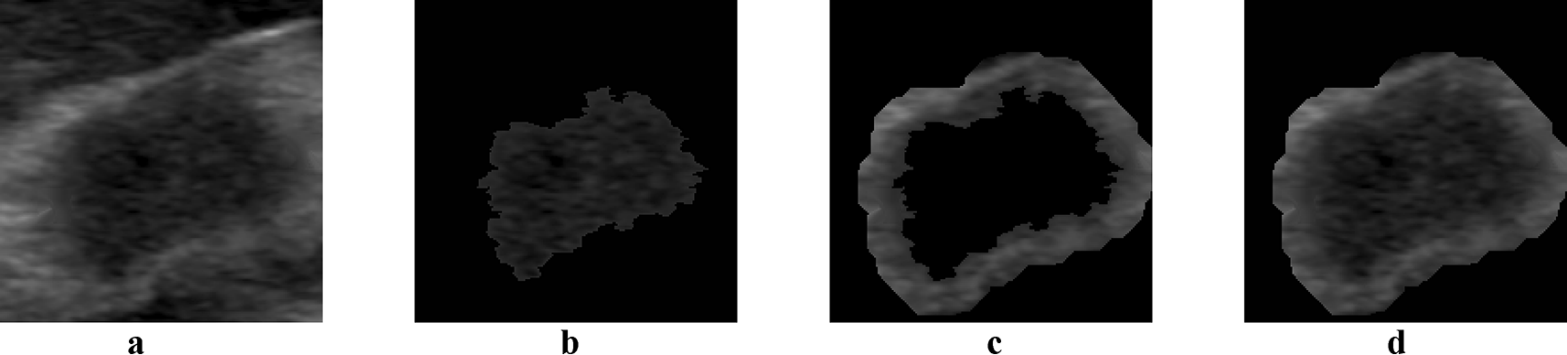
ROIs extracted from the cleaned-up image: (**a)** original ROI, **(b)** intra-tumoral ROI, **(c)** peritumoral ROI and **(d)** combined ROI.

Subsequently, four different frames containing the ROI were extracted from each cleaned-up image. In Fig. 3, an example is depicted. The first image corresponds to the bounding box containing both the intra-tumoral and the peritumoral region. The second image was automatically segmented implementing an inhouse region growing algorithm with the aim of isolating the intra-tumoral region. Starting from a center pixel, this technique allows to iteratively dilate the region of interest comparing pixels in the region with a neighboring one by means of a similarity measure, defined as the difference between the gray-level value of this candidate and the gray-level mean value of the area^26^. Then, on the intra-tumoral ROI, an erosion technique was applied for obtaining the fourth image. This procedure permits to expand the input object by a specified measure, without modifying the image contour^27^. In this study, a 2 cm dilation was performed, according to our radiologist’s directives. Finally, the third image containing only the peritumoral region was obtained as the combined and intra-tumoral ROI subtraction.

### Radiomic feature extraction

From each of the four previously pre-processed ROIs, 134 radiomic features were automatically extracted to describe the images texture. Basically, four different matrices were computed: the gray-level co-occurrence matrix (GLCM), the gray-level run length matrix (GLRLM), the gray-level size zone matrix (GLSZM) and the neighborhood gray-tone difference matrix (NGTDM). The statistical measures extracted from the GLCM characterize the image texture estimating how often pairs of pixels with specific values and in a specified spatial relationship occur in the image itself^28^. An account of the gray level runs, defined as the length in number of consecutive pixels characterized by the same gray level value, is given by the statistical features computed on the GLRLM^29^. The statistical measures extracted from the GLSZM allow to describe the amount of gray level zone, that are the number of connected areas that share the same gray level intensity^30^. Finally, a measure of the difference between the gray value of each pixel and the average gray value of its neighbors is given by the statistical features computed on the NGTDM^31^.

Radiomic analysis was performed by means of Matlab toolboxes^32–35^, setting free parameters with default values and computing the matrices in the four possible directions ( 0°, 45°, 90°, 135°).

### Statistical analysis

With the aim of evaluating the contribution of each clinical feature on the ALN metastatic status, a preliminary statistical analysis was performed by means of the Mann–Whitney test for variables measured on a continuous scale and the Chi-square test for variables measured on a nominal scale. A feature was considered statistically significant if the performed statistical test returned a *p*-value less than 0.05. Subsequently, in order to ensure homogeneity between the training and test sets, a further statistical analysis was implemented to compare the distributions of both sub-sets with respect to all clinical features involved in this study. Two distributions were considered statistically different if the statistical test returned a *p*-value less than 0.05.

### Learning model

**Figure 4:**
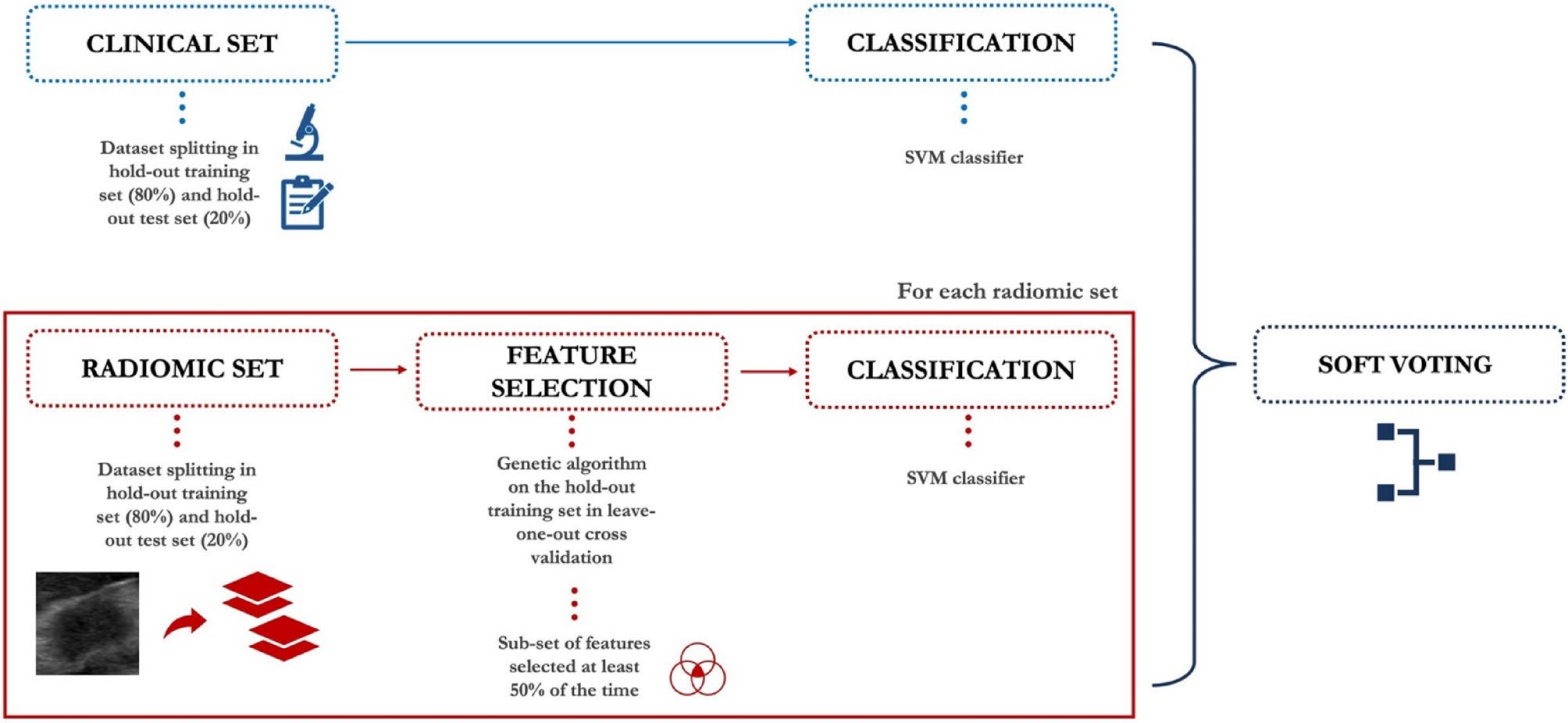
Schematic overview of the proposed approach. First, clinical and radiomic features were evaluated separately by implementing two different machine learning approaches. Subsequently, a soft voting technique was performed between the clinical-based model and each radiomic-based model.

By means of the features collected in the previous steps, eleven different learning models were developed and compared to determine the best one able to predict the ALN metastatic status. Originally, clinical and radiomic features were evaluated separately implementing two different machine learning approaches (*see* Fig. 4). Afterwards, their predictive power was estimated jointly adopting a third machine learning approach, namely soft voting (SV).

All models share both a prior dataset splitting step, by which the original sample is divided in hold-out training set (containing 80% of the sample) and hold-out test set (containing the remaining 20%), and the classification step, based on the support vector machine (SVM) classifier. SVM classifier is a supervised machine learning model which identifies, among all the possible hyperplanes which separate the two classes of data points, the one which has the maximum margin, that is the maximum distance between data points of both classes, by means of a kernel function. For our study the radial basis function was adopted^36^.

Although the clinical-based model consisted only of the data splitting and classification steps, the radiomic-based models required a feature selection stage to reduce the dataset dimension and avoid overfitting. Due to the relatively small dimension of the sample, the feature selection procedure was performed on the hold-out training set in a leave-one-out cross-validation scheme^37^ by means of a genetic algorithm (GA), and different classification models were trained switching, in turns, the subset of features according to the frequency with which these resulted important over the cross-validation rounds. GA is an optimization algorithm based on the genetic theory in biology. As well as in biology stronger individuals have a higher probability to pass on their genes to their children, GA select the optimal combination of features evaluating the probability of different randomly generated populations of being the strongest possible parents^38–40^.

Finally, the SV approach allowed us to examine the mutual influence of the clinical-based model and each of the radiomic-based models in predicting the classification outcome. SV is a machine learning approach belonging to the ensemble methods class. Ensemble methods use the collective judgment of multiple classifiers trained on the same data for making a prediction: every individual classifier provides, for each element, a probability of belonging to a class, and the final prediction can be computed in different ways according to the designated technique. Specifically, SV combines individual predictions averaging the scores attributed by each of the considered model^41–43^.

All the presented models were evaluated in terms of Area Under the Curve (AUC) of the Receiver Operating Characteristic (ROC) curve and other standard metrics such as accuracy, sensitivity and specificity computed setting the threshold value equals to the ratio of the number of patients with positive ALN metastatic status over the total number of patients. Additionally, in order to estimate the variability of these metrics, the 95% confidence intervals were computed on 200 bootstrap rounds with the R library *pROC*.

All the analysis steps were performed by using MATLAB R2021b (Mathworks, Inc., Natick, MA, USA) and R Statistical (v4.1.1., R Core Team 2021) softwares.

### Ethics approval and consent to participate

The study was conducted according to the guidelines of the Declaration of Helsinki, and approved by the Scientific Board of Istituto Tumori ‘Giovanni Paolo II’ (Bari, Italy)—Prot. 6629/21.

### Consent for publication

Informed consent was obtained from all subjects and/or their legal guardian(s).

## Supplementary Information

Supplementary Information.

## References

[1] DeSantis CE (2014). Cancer treatment and survivorship statistics, 2014. CA Cancer J. Clin..

[2] DeSantis CE (2019). Breast cancer statistics, 2019. CA Cancer J. Clin..

[3] Szychta, P. *et al.* Intraoperative diagnosis of sentinel lymph node metastases in breast cancer treatment with one-step nucleic acid amplification assay (OSNA). in *Archives of Medical Science* vol. 12 1239–1246 (Termedia Publishing House Ltd., 2016). 10.5114/aoms.2016.62902 PMC5108387 27904514

[4] Krag UDN (2010). National Surgical Adjuvant Breast and Bowel Project Sentinel-lymph-node resection compared with conventional axillary-lymph-node dissection in clinically node-negative patients with breast cancer: overall survival fi ndings from the NSABP B-32 randomised phase 3 trial. Lancet Oncol..

[5] DiSipio T, Rye S, Newman B, Hayes S (2013). Incidence of unilateral arm lymphoedema after breast cancer: A systematic review and meta-analysis. Lancet Oncol..

[6] Schirosi L (2016). Is immunohistochemistry of BRAF V600E useful as a screening tool and during progression disease of melanoma patients?. BMC Cancer.

[7] Massafra R. *et al.* Decision support systems for the prediction of lymph node involvement in early breast cancer. *J. B.U.ON. Offic. J. Balkan Union Oncol.* (2021). 33721462

[8] Yang J (2019). Preoperative prediction of axillary lymph node metastasis in breast cancer using mammography-based radiomics method. Sci. Rep..

[9] Santucci D (2021). 3T MRI-radiomic approach to predict for lymph node status in breast cancer patients. Cancers (Basel).

[10] Liu J (2019). Radiomics analysis of dynamic contrast-enhanced magnetic resonance imaging for the prediction of sentinel lymph node metastasis in breast cancer. Front. Oncol..

[11] Qiu X (2020). Could ultrasound-based radiomics noninvasively predict axillary lymph node metastasis in breast cancer?. J. Ultrasound Med..

[12] Zhou WJ, Zhang YD, Kong WT, Zhang CX, Zhang B (2021). Preoperative prediction of axillary lymph node metastasis in patients with breast cancer based on radiomics of gray-scale ultrasonography. Gland Surg..

[13] Sun Q (2020). Deep learning vs. radiomics for predicting axillary lymph node metastasis of breast cancer using ultrasound images: Don’t forget the peritumoral region. Front. Oncol..

[14] Zheng X (2020). Deep learning radiomics can predict axillary lymph node status in early-stage breast cancer. Nat. Communications.

[15] Dihge L, Ohlsson M, Edén P, Bendahl PO, Rydén L (2019). Artificial neural network models to predict nodal status in clinically node-negative breast cancer. BMC Cancer.

[16] Fanizzi A (2021). Predicting of sentinel lymph node status in breast cancer patients with clinically negative nodes: A validation study. Cancers (Basel).

[17] Fanizzi A (2021). Sentinel lymph node metastasis on clinically negative patients: Preliminary results of a machine learning model based on histopathological features. Appl. Sci..

[18] Fanizzi A (2021). Advancement study of CancerMath model as prognostic tools for predicting Sentinel lymph node metastasis in clinically negative T1 breast cancer patients. JBUON.

[19] Amoroso N (2015). Hippocampal unified multi-atlas network (HUMAN): Protocol and scale validation of a novel segmentation tool. Phys. Med. Biol..

[20] Quail DF, Joyce JA (2013). Microenvironmental regulation of tumor progression and metastasis. Nat. Med..

[21] Yi A (2013). Association of tumour stiffness on sonoelastography with axillary nodal status in T1 breast carcinoma patients. Eur. Radiol..

[22] Lambin P (2017). Radiomics: The bridge between medical imaging and personalized medicine. Nat. Rev. Clin. Oncol..

[23] Gatta G (2021). Second-generation 3D automated breast ultrasonography (Prone ABUS) for dense breast cancer screening integrated to mammography: Effectiveness, performance and detection rates. J. Pers. Med..

[24] Criminisi, A., Pérez, P. & Toyama, K. *Region Filling and Object Removal by Exemplar-Based Image Inpainting*. *IEEE TRANSACTIONS ON IMAGE PROCESSING* vol. 13 www.csse.monash.edu.au/ (2004). 10.1109/tip.2004.833105 15449582

[25] Bornemann, F. & März, T. *FAST IMAGE INPAINTING BASED ON COHERENCE TRANSPORT*.

[26] Mancas, M., Gosselin, B. & Macq, B. *Segmentation Using a Region Growing Thresholding*.

[27] Tambe, sagar B., Kulhare, D., Nirmal, M. D., Prajapati, G. & Pune, M. *International Journal of Emerging Technology and Advanced Engineering Image Processing (IP) Through Erosion and Dilation Methods*. *Certified Journal* vol. 9001 www.ijetae.com (2008).

[28] Haralick RR, Shanmugam K (1973). Textural features for image classification. IEEE Trans. Syst. Man Cybern..

[29] Galloway MM (1975). Texture analysis using gray level run lengths. Comput. Graph Image Process.

[30] Thibault, G. *et al. Texture Indexes and Gray Level Size Zone Matrix Application to Cell Nuclei Classification*.

[31] Mayerhoefer ME (2020). Introduction to radiomics. J. Nucl. Med..

[32] Avinash Uppuluri. GLCM texture features. (2021).

[33] Xunkai Wei. Gray Level Run Length Matrix Toolbox. (2021).

[34] Martin Vallieres. Gray Level Size Zone Matrix Toolbox. (2021).

[35] Abhijith Bailur. Neighborhood gray-tone difference matrix Toolbox. (2021).

[36] Cyran K.A. & et al. Support vector machines in Biomedical and Biometrical Applications. in *Emerging Paradigms in Machine Learning Smart Innovation, Systems and Technologies,* 13 (2013).

[37] Cawley et al. Efficient leave-one-out cross-validation of kernel fisher discriminant classifiers. *Pattern Recognition*.

[38] Too J, Abdullah AR, Saad NM, Tee W (2019). EMG feature selection and classification using a Pbest-guide binary particle swarm optimization. Computation.

[39] Huang CL, Wang CJ (2006). A GA-based feature selection and parameters optimizationfor support vector machines. Expert Syst. Appl..

[40] Goldberg_Genetic_Algorithms_in_Search.

[41] Polikar R (2006). Ensemble based systems in decision making. IEEE Circuits Syst. Mag..

[42] Sagi, O. & Rokach, L. Ensemble learning: A survey. *Wiley Interdisciplinary Reviews: Data Mining and Knowledge Discovery* vol. 8 (2018).

[43] Sewell, M. & Tat, R. Ensemble learning Related papers Ensemble Met hods Mart in Sewell T he Superiorit y of t he Ensemble Classificat ion Met hods: A Comprehensive Review Nzuva M Silas Classifier Combinat ion for In Vivo Magnet ic Resonance Spect ra of Brain Tumours Ensemble Learning Ensemble Learning. (2011).

