# Supplementary material for: A ultrasound-based radiomic approach to predict the nodal status in clinically negative breast cancer patients
Source: Sci Rep. 2022 May 12;12:7914. doi: 10.1038/s41598-022-11876-4 (PMC9098914; doi:10.1038/s41598-022-11876-4)
Supplement: Supplementary file 1 — Supplementary Information. [file 41598_2022_11876_MOESM1_ESM.docx]

**Supplementary Tables**

**Table S1. Comparison among clinical features distributions over the hold-out training and hold-out test sets.** The asterisk * highlights features with a *p*-value less than 0.05. The statistical analysis was performed by means of the Mann-Whitney test for variables measured on a continuous scale and the Chi-square test for variables measured on a nominal scale.

| **Feature** | **Training test** | **Test set** | **Feature** | **Training test** | **Test set** |
| --- | --- | --- | --- | --- | --- |
| **Age** median; [q_1_, q_3_] | 59; [48,68] | 64; [44,69] | **Quadrant** |  |  |
| **Diameter** |  |  | QSM (abs.; %) | 17 - 14.9% | 3 - 10.7% |
| T1a (abs.; %) | 10 - 8.8% | 2 - 7.1% | QSE (abs.; %) | 34 - 29.8% | 12 - 42.8% |
| T1b (abs.; %) | 35 - 30.7% | 8 - 28.6 % | QEE (abs.; %) | 19 - 16.7% | 1 - 3.6% |
| T1c (abs.; %) | 43 - 37.7% | 14 - 50 % | QIE (abs.; %) | 14 - 12.3% | 1 - 3.6% |
| T2 (abs.; %) | 26 - 22.8% | 4 - 14.3% | QIM (abs.; %) | 1 - 0.8% | 0 - 0% |
| **Grading** |  |  | QII (abs.; %) | 6 - 5.3% | 2 - 7.1% |
| G1 (abs.; %) | 34 - 29.8% | 8 - 28.6% | QEI (abs.; %) | 5 - 4.4% | 4 - 14.3% |
| G2 (abs.; %) | 50 - 43.9% | 12 - 42.8% | QSI (abs.; %) | 18 - 15.8% | 5 - 17.9% |
| G3 (abs.; %) | 30 - 26.3% | 8 - 28.6% | **Her2/neu** |  |  |
| **Histological type** |  |  | 0 (abs.; %) | 73 - 64.1% | 15 - 53.6% |
| Ductal (abs.; %) | 93 - 81.6% | 23 - 82.1% | 1 (abs.; %) | 25 - 21.9% | 6 - 21.4% |
| Lobular (abs.; %) | 16 - 14% | 5 - 17.9% | 2 (abs.; %) | 9 -7.9% | 4 - 14.3% |
| Others (abs.; %) | 5 - 4.4% | 0 - 0% | 3 (abs.; %) | 7 - 6.1% | 3 - 10.7% |
| **Invasiveness** |  |  | **Multifocality** |  |  |
| Infiltrating (abs.; %) | 107 - 93.9% | 24 - 85.7% | Absent (abs.; %) | 90 - 78.9% | 20 - 71.4% |
| In situ (abs.; %) | 7 - 6.1% | 4 - 14.3% | Present (abs.; %) | 24 - 21.1% | 8 - 28.6% |
| **Angioinvasion** |  |  | **ki67*** median; [q_1_, q_3_] | 18 [12,30] | 20 [14,30] |
| Absent (abs.; %) | 99 - 86.8% | 22 - 78.6% | **ER*** median; [q_1_, q_3_] | 98 [95,100] | 95 [90,98] |
| Present (abs.; %) | 15 - 13.2% | 6 - 21.4% | **PgR** median; [q_1_, q_3_] | 40 [5,85] | 35 [5,70] |

**Table S2. Classification performances of all models on the hold-out training set.** For each radiomic feature set, the performances of both the related radiomic-based model and the soft voting-based model are reported.

|  | **AUC (%)** | **Accuracy (%)** | **Sensitivity (%)** | **Specificity (%)** |
| --- | --- | --- | --- | --- |
| Clinical | 78.7 | 86.7 | 81.8 | 92.1 |
| Radiomic original | 77.9 | 91.3 | 86.3 | 92.1 |
| Clinical/Radiomic original (SV) | 94.4 | 86.8 | 90.9 | 84.4 |
| Radiomic intra | 72.4 | 78.1 | 68.6 | 80.4 |
| Clinical/Radiomic intra (SV) | 69.8 | 71.8 | 66.3 | 74.5 |
| Radiomic peri | 73.9 | 82.1 | 68.6 | 84.4 |
| Clinical/Radiomic peri (SV) | 79.9 | 78.1 | 68.6 | 80.4 |
| Radiomic comb | 75.1 | 78.5 | 70.4 | 80.4 |
| Clinical/Radiomic comb (SV) | 81.6 | 80.3 | 86.3 | 74.5 |
| Radiomic intra + peri | 71.2 | 82.1 | 68.6 | 84.4 |
| Clinical/Radiomic intra + peri (SV) | 88.3 | 85.2 | 86.3 | 84.4 |

**Table S3. Classification performances of models exploiting both clinical and radiomic features extracted from the original ROI on the hold-out test set.** Whereas the *Clinical/Radiomic original* *(SV)* model combined information provided by both clinical and radiomic features by means of the soft-voting technique, the *Clinical+Radiomic original* model directly combined clinical and radiomic features.

|  | **AUC (%)** | **Accuracy (%)** | **Sensitivity (%)** | **Specificity (%)** |
| --- | --- | --- | --- | --- |
| Clinical+Radiomic original | 59.1 | 75 | 40 | 82.6 |
| **Clinical/Radiomic original (SV)** | **88.6** | **82.1** | **100** | **78.2** |
